# An improved method for preparing Agrobacterium cells that simplifies the Arabidopsis transformation protocol

**DOI:** 10.1186/1746-4811-2-16

**Published:** 2006-10-24

**Authors:** Elke Logemann, Rainer P Birkenbihl, Bekir Ülker, Imre E Somssich

**Affiliations:** 1 Max-Planck-Institute for Plant Breeding Research, Department of Plant Microbe Interactions, Carl-von-Linné-Weg 10, D-50829 Cologne, Germany; 2 Current address: School of Biological and Biomedical Sciences, Durham University, Science Site, South Road, Durham DH1 3LE, UK

## Abstract

**Background:**

The *Agrobacterium *vacuum (Bechtold *et al *1993) and floral-dip (Clough and Bent 1998) are very efficient methods for generating transgenic Arabidopsis plants. These methods allow plant transformation without the need for tissue culture. Large volumes of bacterial cultures grown in liquid media are necessary for both of these transformation methods. This limits the number of transformations that can be done at a given time due to the need for expensive large shakers and limited space on them. Additionally, the bacterial colonies derived from solid media necessary for starting these liquid cultures often fail to grow in such large volumes. Therefore the optimum stage of plant material for transformation is often missed and new plant material needs to be grown.

**Results:**

To avoid problems associated with large bacterial liquid cultures, we investigated whether bacteria grown on plates are also suitable for plant transformation. We demonstrate here that bacteria grown on plates can be used with similar efficiency for transforming plants even after one week of storage at 4°C. This makes it much easier to synchronize *Agrobacterium *and plants for transformation. DNA gel blot analysis was carried out on the T_1 _plants surviving the herbicide selection and demonstrated that the surviving plants are indeed transgenic.

**Conclusion:**

The simplified method works as efficiently as the previously reported protocols and significantly reduces the workload, cost and time. Additionally, the protocol reduces the risk of large scale contaminations involving GMOs. Most importantly, many more independent transformations per day can be performed using this modified protocol.

## Background

*Arabidopsis thaliana *has been the workhorse of plant molecular biologists and is routinely transformed using *Agrobacterium*-mediated transformation methods. The first simple Arabidopsis transformation method reported by Bechtold *et al *(1993) was simplified by Clough and Bent (1998), who eliminated the vacuum-infiltration step. Instead, the authors used floral dipping of Arabidopsis plants into sucrose solution containing the *Agrobacterium *cells in the presence of surfactants. Clough and Bent (2002) tested several other variables also thought to be important for the efficiency of transformation. The authors found that the MS salts, hormones and other constituents make no difference, that the density of the bacteria is not critical, and again that use of vacuum basically has no effect on transformation efficiency as long as a certain amount of surfactant is present. However, plant health and the developmental state of the flowers were found to be the most important factors (Desfeux et al., 2002).

In our laboratory, we had difficulties growing large cultures of the *Agrobacterium *strain GV3101 pMP90RK after transformation with pPAM based vectors [GenBank: AY027531]. The strain transformed with this vector requires selection with three to four different antibiotics and growth on YEB solid and liquid media takes about 3 days. Large liquid cultures from individual colonies, necessary for the plant transformation procedure, often did not grow sufficiently even after four days culturing in flasks on shakers at 28°C. Although this problem usually can be solved by employing small starter cultures, the bacterial growth remained unreliable. We observed that traces of residual detergents or chemicals remaining on flasks or glass tube surfaces after washing were sometimes detrimental for bacterial growth, and bacteria grew much better in sterile Falcon tubes. Additionally, because of limited shaker capacity, we could only transform a limited number of constructs into plants at one given time. Due to the problems encountered, we often missed the optimal developmental stage of plants for transformation and sometimes had to postpone experiments until new plants became available.

Here we describe a modified version of the *Agrobacterium*-mediated transformation that does not require growth of large bacterial liquid cultures. Instead, plants are transformed with bacteria grown on plates. We demonstrate that this method is as efficient and reliable as the previous methods in generating transgenic plants.

## Results and discussion

### Transformation of Arabidopsis plants and BASTA selection

**Figure 1:**
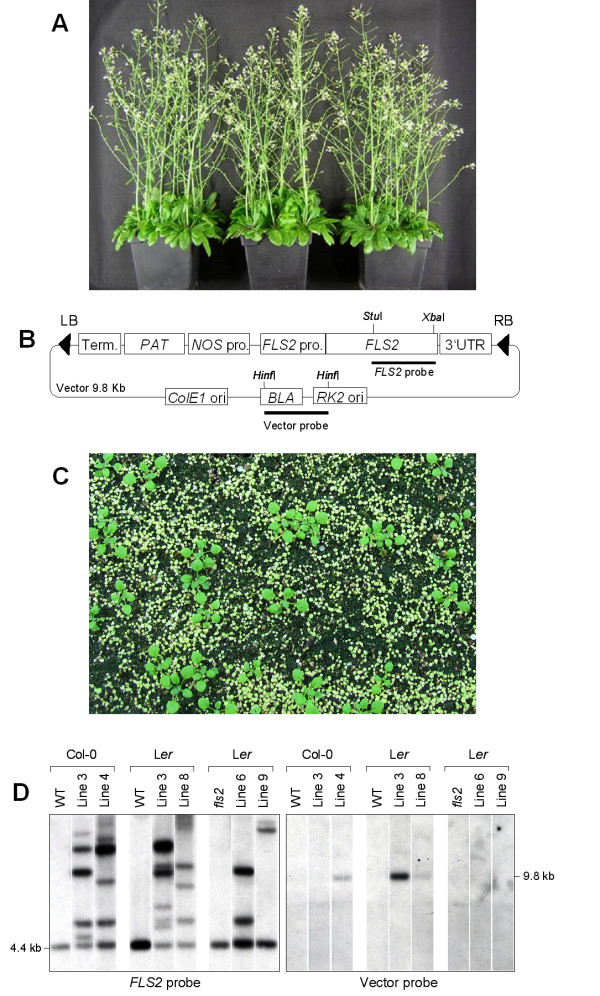
**Selection and molecular confirmation of transgenic Arabidopsis plants**. A. Arabidopsis plants used for floral dip transformations; B. DNA construct used for transformation; LB: left border, RB: right border, *FLS2*: flagellin sensing locus 2, *PAT*: phosphinothricin acetyltransferase conferring BASTA resistance, NOS: nopaline synthase, pro: promoter, *BLA*: β-lactamase conferring ampicillin resistance, ori: origin of replication, UTR: untranslated region, term: terminator. C. Surviving plants following 2 weeks of BASTA selection; D. DNA gel blot analysis: Plant genomic DNA from transformed and non transformed plants (WT, *fls*) from different genetic backgrounds (Col, L*er*, L*er fls*) was digested with *Stu*I, the fragments electrophoretically separated and blotted onto a nylon membrane. The blot was probed with the *Stu*I/*Xba*I fragment of the *FLS2 *coding sequence (left panel) and, after stripping, reprobed with the *Hinf*I fragment of the vector backbone (right panel). 4.4 kb indicates the size of the endogenous *FLS2 Stu*I fragment, while 9.8 kb corresponds to the full size vector DNA digested with *Stu*I.

The Arabidopsis *FLS2 *gene (At5g46330) including its promoter and terminator sequences was cloned into the pPAM [GenBank: AY027531] based pKWS05 vector (Figure 1B). This construct was transformed into two different Arabidopsis ecotypes, Col-0 and L*er*, as well as into Arabidopsis *fls2 *mutant plants previously generated by EMS mutagenesis (Gomez-Gomez and Boller, 2000). In order to avoid the problems associated with growth of bacteria in liquid cultures, we employed bacteria cultured on plates for direct transformation of Arabidopsis plants. After two days of growth under selection, the bacteria were scraped from the plates and resuspended in 30 ml of YEB medium in Falcon tubes. From one densely grown plate this resulted in an OD_600 _of about 2.0. For dipping plants, the bacterial cell suspensions were poured into 2 liter disposable plastic autoclave bags containing 120 ml of 5% sucrose solution supplemented with 0.03% Silwet L-77. Flowers of healthy Arabidopsis plants were subsequently dipped into the bacterial solution contained in the plastic bags. The bacteria were distributed to all plant parts including very young flower shoots by gently pressing the outer side of the bags by ones hands. After ten seconds the plants were removed from the plastic bags and treated as described below (see Methods). Transformed plants were kept in the greenhouse and seeds were harvested upon full maturation. The seeds were germinated on soil and transgenic plants were selected by spraying with 0.1% BASTA herbicide in the greenhouse (Figure 1C). Spraying was performed one week after germination and repeated four times at two-day intervals. Transgenic plants were readily identified at the end of the BASTA selection. While such plants continued to grow and remained green, the untransformed plants remained small, became white and died two weeks after selection (Figure 1C).

By this modified protocol we obtained more than 100 transgenic plants per transformation by dipping two pots each containing 5–8 Arabidopsis plants. This efficiency is highly satisfactory for most applications. Storage of bacteria on plates at 4°C for up to one week did not noticeably reduce the transformation efficiency since the number of transformants obtained using such bacteria were nearly the same when compared with liquid grown cultures. Thus, the method therefore allowed us to even wait for the best suitable developmental stage of plants prior to transformation. The detailed modified transformation protocol is given in the Methods section.

### DNA gel blot analysis confirms successful transformation

In order to prove at the molecular level that the plants surviving the BASTA selection are indeed transgenic, we performed DNA gel blot analysis on these plants and compared the results to the wild type originators. The membrane containing the *Stu*I digested genomic DNAs of selected lines was first hybridized with a 3' *FLS2 *probe (1.5 kb *Stu*I/*Xba*I fragment of the *FLS2 *gene, see Figure 1B) and, after stripping, rehybridized with a vector probe (the 1.5 kb *Hinf*I fragment of pKWS05). The results are shown in Figure 1D. As expected, the *FLS2 *probe only detected a 4.4 kb *Stu*I fragment corresponding to the endogenous *FLS2 *gene in the wild type plants and the *fls2 *mutant (EMS mutant). In the transgenic plants, beside the 4.4 kb band additional bands could be observed. To eliminate the possibility that the additional bands might be the due to the presence of contaminating *Agrobacterium *in these plants, we reprobed the membrane with the *Hinf*I fragment from the vector backbone, which should not be inserted into the plant genome (Figure 1D right panel). If intact *Agrobacterium *DNA was isolated together with the plant DNA, a 9.8 kb band corresponding to the size of the linearized vector is expected and should be detected. Although we detected contaminating *Agrobacterium *DNA in Col line 4, and in L*er *lines 3 and 8 (Figure 1D), no other bands were visible even after very long exposure times (data not shown). Therefore, the other additional bands observed with the *FLS2*-probe are derived from stably integrated copies of the *FLS2 *gene introduced by the T-DNA. This is strong evidence that these plants are true transgenics.

### Other examples of successful transformations

Using this modified transformation method we transformed four other constructs into wild type or T-DNA mutant plants. These constructs contained cDNAs coding for four different *WRKY *transcription factors carrying HA epitope tags at their C-terminal ends. The constructs were transformed into both wild type Col-0 plants as well as the corresponding homozygous *WRKY *T-DNA mutant lines. Also in these cases, we obtained more than 100 transformants surviving the BASTA selection. In addition, we could detect the production of the recombinant proteins in about 30% of the T_1 _plants by western analysis using an anti-HA antibody (data not shown).

Our data demonstrate that the method is suitable for transforming different Arabidopsis ecotypes, such as Col-0 and L*er *as well as mutant plants such as the *fls2 *mutant. We also successfully applied this method to generate a similar set of *FLS2 *transgenic lines using another binary vector that allows for selection with the antibiotic kanamycin (data not shown).

## Conclusion

Our protocol eliminates the risk of large scale contaminations involving GMOs, reduces the volume of sucrose solution and the amount of sucrose needed. Furthermore, it prevents the need for large flasks and centrifuge bottles that require further cleaning and decontamination. The improvements made to the transformation method allowed us to omit several time consuming and equipment requiring steps such as shaking of large bacterial cultures at 28°C for two to three days, several centrifugation steps and use of large glassware like flasks and beakers. Thus, the method saves media, antibiotics, sucrose, electricity, and no glassware has to be washed. Although this method was only tested using Arabidopsis ecotypes and mutants, we see no reason why this method should not also be suitable for transformation of other plants for which the floral dip method has been successfully applied.

## Methods

### The simplified transformation method modified from the protocol by Clough and Bent

- Grow healthy Arabidopsis plants until they are flowering (see Figure 1A).

Optional: Clip first bolts to encourage proliferation of many secondary bolts. Plants will be ready roughly 4–6 days after clipping. Optimal plants have many immature flower clusters and only few fertilized siliques, although a range of plant stages can be successfully transformed.

- Transform the DNA construct of interest into the appropriate *Agrobacterium *strain. Grow the transformed *Agrobacterium *on YEB (or LB) plates containing the appropriate antibiotics in a 28°C incubator.

- Select a colony and resuspend bacteria in 10 μl H_2_O. Plate half of the volume immediately as a lawn onto a YEB plate with the suitable antibiotics and incubate at 28°C for (2–3 days), and use the other half to verify the presence of your DNA construct by PCR analysis.

- Collect the densely grown bacteria from the plate by scraping, and resuspend them in 30 ml YEB in a sterile Falcon tube. The OD_600 _should be about 2.0.

- Per transformation prepare 120 ml of 5% sucrose solution containing 0.03% of Silwet L-77 (surfactant; Lehle Seeds), pour solution into a disposable plastic bag and add the bacteria.

- Dip the inflorescences of the plants into the *Agrobacterium *solution for 10 seconds, under gentle agitation. You should observe a film of liquid coating the plants. The bacteria are distributed to all plant parts including very young flower shoots by gently pressing the outside of the bag with your hands.

- Place dipped plants under a lid or cover for 16 to 24 hours to maintain high humidity (plants can be laid on their sides if necessary). Do not expose to excessive sunlight (the temperature under the lid can get high).

- Water and grow the plants as normal, tying up loose bolts with wax paper, tape, stakes, twist-ties, or by other means. Stop watering as seeds become mature.

- Harvest dry seeds.

- Select for transformants using appropriate antibiotics or herbicides.

### DNA gel blot analysis

DNA was isolated from 150 mg plant leaf material using the Nucleon Phytopure Kit of Amersham (catalogue no. RPN8510). 10 μg genomic DNA from each line was digested with *Stu*I and separated on a 0.8% agarose gel. The gel was denatured and neutralised and the DNA was transferred to a nylon membrane and hybridized with the ^32^P-labelled 3' *FLS2 *probe and vector probes. Labelling was performed using the Random Prime DNA Labelling Kit of Roche (catalogue no. 1004760). Hybridization was carried out overnight in a buffer containing 1 M NaCl, 1% SDS, 10% Dextran sulphate and 200 μg/ml salmon sperm DNA. The membrane was washed with 0.2 × SSC, 0.5% SDS at 68°C. For stripping the filter, the membrane was washed in 0.1% SDS solution for 45 min at 94°C.

## Competing interests

The author(s) declare that they have no competing interests.

## Authors' contributions

EL, RPB and BÜ conceived and designed the experiments reported in this study. EL and RPB carried out the transformations and molecular analyses and helped in drafting the manuscript. IES participated in drafting the final manuscript. BÜ drafted the manuscript and coordinated the study together with IES. All authors read and approved this final version of the manuscript.
